# A nanowell platform to identify, sort and expand high antibody-producing cells

**DOI:** 10.1038/s41598-024-60054-1

**Published:** 2024-04-24

**Authors:** Fikri Abali, Richard Schasfoort, Sanne Nijland, Jelle Wittenberns, Arjan. G. J. Tibbe, Marcel den Hartog, Louis Boon, Leon W. M. M. Terstappen

**Affiliations:** 1 Department of Medical Cell BioPhysics, Faculty of Science and Technology, University of Twente, PO Box 217, 7500AE Enschede, The Netherlands; 2 VYCAP, Capitool 41, 7521PL Enschede, The Netherlands; 3 Polpharma Biologics Utrecht B.V., Yalelaan 46, 3584 CM Utrecht, The Netherlands; 4 JJP Biologics, Bobrowiecka 6, 00-728 Warsaw, Poland; 5 Department of General, Visceral and Pediatric Surgery, Heinrich-Heine University, University Hospital Düsseldorf, 40225 Düsseldorf, Germany

**Keywords:** Nanowell chip, CHO cells, Single-cell analysis, Antibody secretion, Clonal expansion, Biological techniques, Biotechnology

## Abstract

Increased use of therapeutic monoclonal antibodies and the relatively high manufacturing costs fuel the need for more efficient production methods. Here we introduce a novel, fast, robust, and safe isolation platform for screening and isolating antibody-producing cell lines using a nanowell chip and an innovative single-cell isolation method. An anti-Her2 antibody producing CHO cell pool was used as a model. The platform; (1) Assures the single-cell origin of the production clone, (2) Detects the antibody production of individual cells and (3) Isolates and expands the individual cells based on their antibody production. Using the nanowell platform we demonstrated an 1.8–4.5 increase in anti-Her2 production by CHO cells that were screened and isolated with the nanowell platform compared to CHO cells that were not screened. This increase was also shown in Fed-Batch cultures where selected high production clones showed titers of 19–100 mg/L on harvest day, while the low producer cells did not show any detectable anti-Her2 IgG production. The screening of thousands of single cells is performed under sterile conditions and the individual cells were cultured in buffers and reagents without animal components. The time required from seeding a single cell and measuring the antibody production to fully expanded clones with increased Her-2 production was 4–6 weeks.

## Introduction

Monoclonal antibodies (mAbs) have become an important group of biologics in modern medicine^1^. The increasing importance of therapeutic mAbs is apparent as mAbs have become the predominant treatment of cancer and autoimmune diseases^2,3^.

The successful discovery of antibodies for therapies requires the implementation of efficient antibody discovery and cell line development methodologies. These methods are crucial for addressing several key challenges, including screening large cell populations for productivity, antigen specificity, and other essential parameters^4,5^. In addition, a major hurdle is the isolation of the rare cell that produces the right antibody in high concentrations while assuring monoclonality^6–9^. Production of mAbs using Chinese hamster ovary (CHO) cells, is the most common method for expression and mass production of therapeutic mAb^10^ and various guidelines are available to assure a safe and reliable production^11^. mAb-producing CHO cells are obtained by inserting a specific gene encoding the antibody of interest into the CHO cells which then produce and secrete the antibody. Although, all cells will have the same gene built in and should produce the same amount of mAbs. However, due to gene loss and/or rearrangement, or other uncertain mechanisms, some CHO cells will inevitably lose their ability to secrete mAb during the culture process even when derived from a single progenitor^12–14^. This phenomenon causes the mAb production of a population to become inefficient resulting in higher costs in reagents and labor. The production of mAbs will also be further reduced if the population of high producing CHO cells is outnumbered by the population of low producing cells because they have a greater growth potential compared to the high producing CHO cells. To produce high titers of mAbs, it is therefore important to screen CHO cell pools to select the high-producing ones. The most traditional method to screen high-producing CHO cells is based on limiting dilution. This involves seeding CHO cells in 96 well plates to obtain monoclonal populations^15,16^. For screening a heterogeneous population of CHO cells in mAb production with limiting dilution, CHO cells are diluted to 3 ~ 5 cells/ml and seeded in 96 well plates resulting in 0.3–0.5 cell/well. Next, the wells are monitored for identify wells with single cells and these are cultured for 7–14 days followed by determining the secreted amount of mAb, using for example ELISA. The wells that do contain the high producers of mAbs are further cultured. This limiting dilution sequence is mostly repeated multiple times. Although limiting dilution can provide stable clones with a high probability of being monoclonal, that produce mAbs in the highest quantity, the method is lengthy, labor-intensive, and consumes a lot of reagents, and multiple rounds of screening are required to obtain stable clones^15,16^.

For this reason, continuous exploration is underway to enhance methods and technologies for clonal selection, antibody discovery, and cell line development. Technologies like Immunospot ^17–19^, droplet-based microfluidics ^18,20^, and micro- or nanowell technologies^21^ have emerged for these purposes. While these technologies exhibit promise, they also present certain limitations. Single-cell handling and retrieval can be challenging, ensuring clonality is difficult, dedicated reagents need to be developed, or incur significant costs.

Here we use the nanowell platform that uses an array of 6400 wells for single seeding and isolation. Each nanowell chip can hold a single cell that is confirmed by acquiring images of each well^22^. The antibody production produced by a single cell that resides inside the nanowell is determined using common ELISA based analysis. The single cells that produce the antibody against the target of interest in the highest amount are transferred and expanded for antibody production^23,24^. The performance of this nanowell platform is demonstrated using CHO cells producing IgG antibodies against Her-2.

## Results

### Cell selection based on antibody production

**Figure 1:**
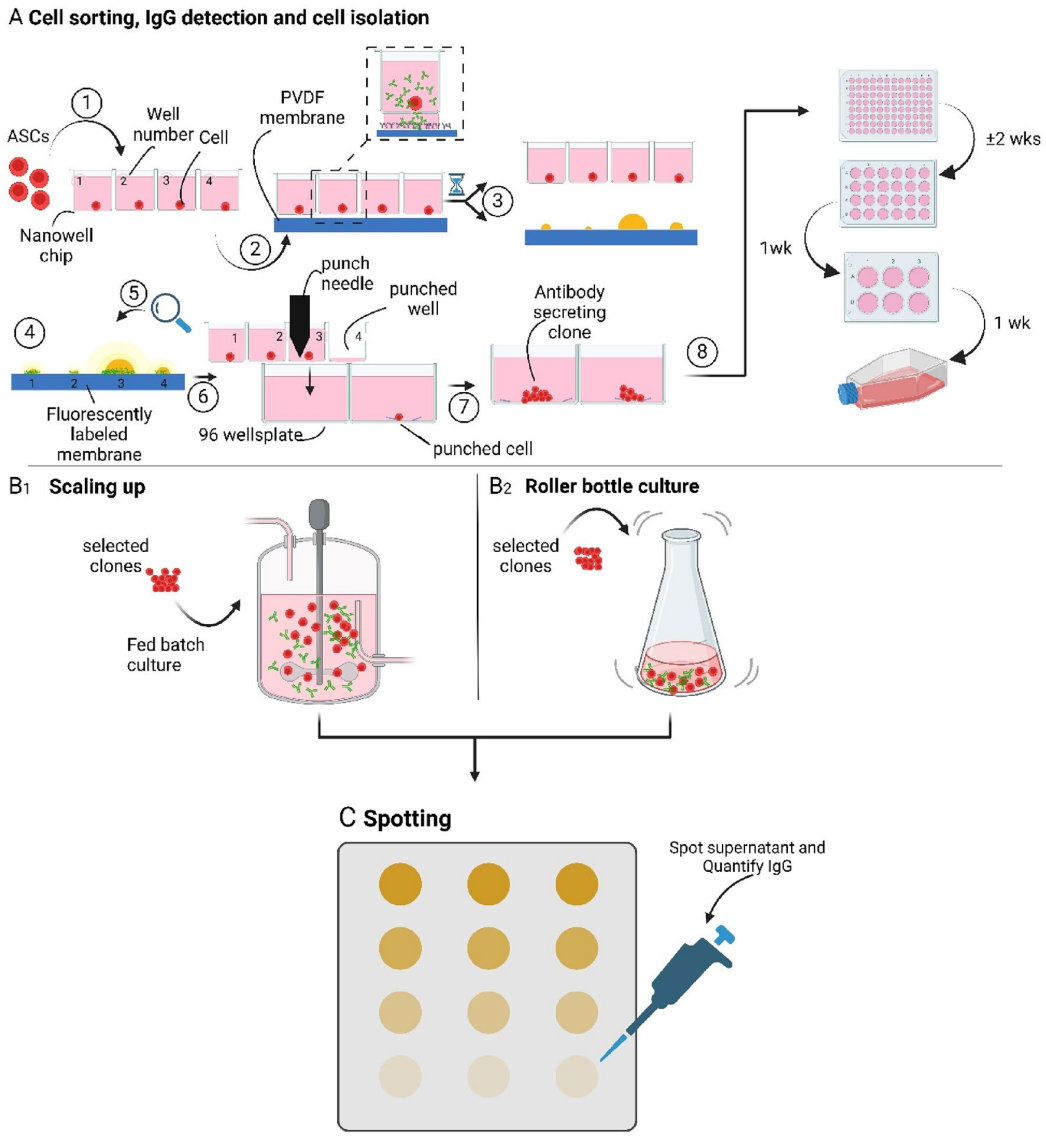
Schematic presentation of the nanowell platform to identify, sort and expand high antibody-producing cells. (**A**) Antibody-secreting cells (ASCs) are sorted in individual wells (1) and connected to an anti-IgG coated PVDF membrane to capture the single-cell secreted anti-Her2 IgG (2) during overnight incubation. The membrane and nanowell device are separated (3) and the membrane is fluorescently labeled to detect the secreted IgGs by the single-cells (CHO cells) to identify high-producing cells (4 and 5). The high-producing cells are isolated from the nanowell device by punching and collecting the cells in individual wells of 96 wells plate (6) for clonal expansion (7). (**B1**) Fed batch cultures in bioreactor and (**B2**) roller bottle culture to determine IgG secretion from clonally expanded high producer cells. (**C**) Supernatant harvested during culture (Fed-batch or roller bottle culture) is spotted on PVDF membrane to determine total and single-cell IgG in time for the selected clones.

Figure 1 depicts the process of CHO cell selection based on their individual anti-Her2 IgG production followed by their isolation and expansion. First, a 1 ml CHO cell suspension containing approximately 5000 cells, is transferred onto a self-sorting nanowell device (1), and a PVDF membrane coated with anti-IgG is placed under the nanowell device (2). The nanowell device connected to a PVDF membrane is placed in the incubator to allow the produced Her-2 antibodies to diffuse through the pore in the bottom of the nanowell and bind to the anti-IgG labeled PVDF membrane. After overnight incubation, the membrane is detached from the nanowell device, and the produced amount of anti-Her 2 antibodies is determined at each nanowell position by fluorescent labelling of the PVDF membrane (3). The nanowell device with the cells is placed back in the incubator awaiting the results of the PVDF staining and analysis (4,5). The location of the anti-Her2 IgG spots on the PVDF membrane is matched to the nanowell array location. Next, the nanowell array is placed on the fluorescence imaging part of the Puncher system, which is located in a laminar flow hood, and a 96-well culture plate, prefilled with culture medium, is loaded on the Puncher system (6). The single CHO cells that are selected based on the produced amount of anti-Her2 IgG are transferred from the nanowell chip towards the 96-well plate by punching the bottom plus cell from the nanowell array towards a well of the prefilled 96-well plate (7). The isolation of 96 cells from the nanowell chip towards individual wells of a 96-well plate takes 5–6 h but in general, the number of high producers is much lower than 96. After isolation, the 96-well plate is placed in the incubator to allow the individual CHO cells to expand (8). The wells of the 96 well plate that show sufficient cell growth are further expanded to create an anti-Her2 IgG-producing cell line which requires 4 weeks. If further selection is needed this above listed process can be repeated. Titers of selected and expanded cells are determined in a fed-batch (B1) or in a roller bottle culture (B2) where the culture supernatant containing the secreted anti-Her2 IgG is collected (at several days) and the amount of produced antibody is quantified using Elispot (C).

### Efficiency of single-cell seeding and conditions to expand single CHO cells after punching

**Figure 2:**
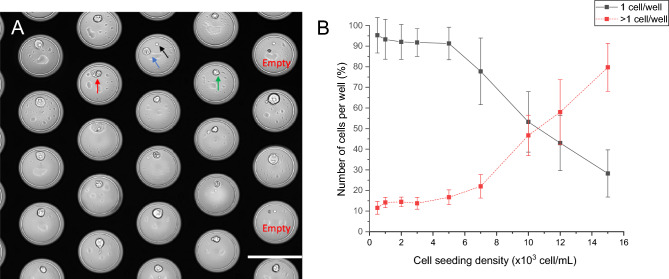
(**A**) CHO cell distribution and morphology after seeding in the nanowell device. The red arrow shows a well with 2 cells, the green arrow a well with a single cell on the pore, the blue arrow a cell off the pore, the black arrow the pore of the well and the well with no cells are denoted with empty. (**B**) The effect of seeding density to obtain an efficient distribution of wells filled with a single cell.

The nanowell device contains 6400 wells each holding a volume of 1.4 nl and each well is closed with an optically transparent bottom that contains a 5 µm pore. During cells seeding fluid passes from the top of the nanowell through the 5 µm pore hereby forcing the cell into the well. Once a single cell lands on the pore, the pore is blocked preventing any other cell from being dragged into the same well. The nanowell array is contained within an area of 8 × 8 mm that is mounted in a holder onto which the cell suspension is deposited. To determine the optimal concentration of cells for cell seeding that provided the largest percentage of wells occupied with single cells a volume of 1 ml containing 500–15,000 CHO cells was placed on the nanowell device. The presence and number of cells in each well was verified by microscopic examination. Figure 2 Panel A shows a brightfield image of a section of the nanowell device. In this section the bottoms of 20 wells are visible. Two wells are empty, 17 contain a single cell and one well indicated with the red arrow show a well with potentially two cells. To maintains monoclonality this well is discarded for further selection and isolation.

The blue arrow indicates a cell that moved from the pore (black arrow) at the time of image acquisition. In the other wells, the cell still is on the pore as indicated by a green arrow in one of the wells. After 24 h of incubation and visual inspection, 63% (± 12%) of the cells remain on the pore while others migrated from the pore. No difference in measured IgG production was observed between cells that remained on the pore and cells that migrated off the pore to a different location on the bottom of the well^23^. The pores in the bottom of the nanowell are off-center to facilitate the “punching” as the needle hits the center of the bottom of the well to catapult the bottom from the well while preventing potential cell damage by this “punching” process. The percentage of wells with a single cell was larger than 90% at cell concentrations ranging from 500 to 5000 cells/ml and dropped at higher concentrations as shown in Fig. 2B. The optimal number of cells presented in 1 ml of loading buffer to obtain a maximum number of wells filled with a single cell was around 5000 cells. While seeding a lower number of cells is possible, this will limit the total amount of cells that can be screened for IgG production. Sterility, medium composition in which the cells reside, and surface characteristics of the nanowell are critical factors to maintain cell viability and optimal conditions for antibody production during incubation. For the anti-Her2 producing CHO cells it was found that a 24h incubation was sufficient to collect a concentration of anti-Her2 antibody on the PVDF membrane that is high enough to detect.

### Quantification of Her-2 production of single CHO cells

**Figure 3:**
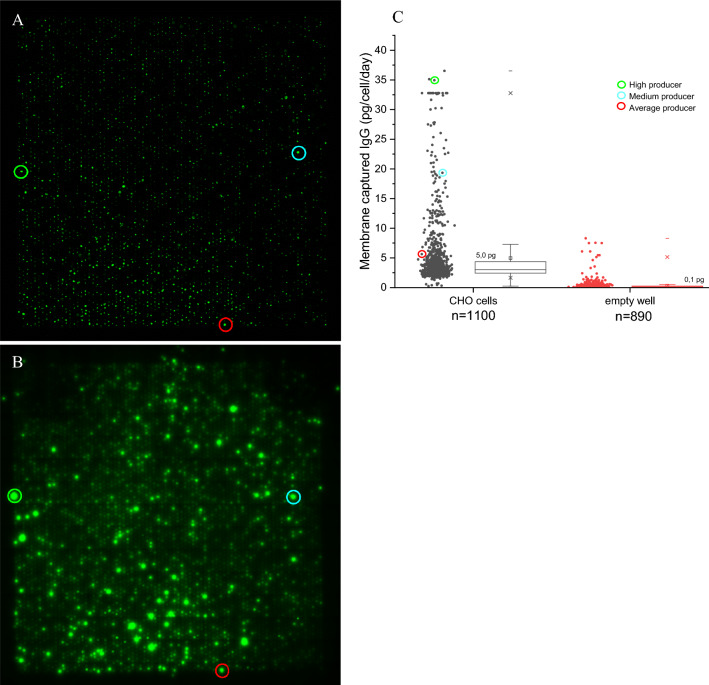
Fluorescence image of the CHO cells inside the nanowells (Panel A), from which 3 cells are highlighted by a in green, blue, and red circle. In panel B shows the corresponding secreted anti Her-2 IgG antibody array captured on the PVDF. Panel C provides the quantitative analysis of the Her-2 production from > 1000 single CHO cells in the nanowell device and the circles matches with the corresponding marked cells in panel A and the Her-2 signal of panel B.

The production of Her-2 by CHO cells was determined after seeding 5000 CHO cells into a nanowell device containing 6400 wells that was placed onto an anti-IgG labelled PVDF membrane, and the Her-2 production was measured after 24 h of incubation. The results of a typical experiment are illustrated in Fig. 3. Panel A shows a fluorescence image of calcian AM labeled CHO cells labelled that are present in the nanowell device after seeding. Out of the 6400 wells, 4549 wells contained a single cell, while the remaining wells contained 2 or more cells or were empty. Panel B presents the PVDF membrane with cell-secreted anti-Her2 IgG antibody that is labeled with anti-IgG PE. Each spot corresponds with the antibody produced by a single CHO cell. The difference in size and intensity of the spots equals the different quantities of the produced anti-Her2 IgG. Panel C presents the production in pg of anti-Her2 IgG produced per day per cell of 1100 randomly selected wells that contained a single cell as well as the measured production level at the location of 890 wells that did not contain a cell. The methodology to quantify the amounts of cell-secreted antibodies captured on the PVDF membrane is described in Abali et al^23^.

In panel A the circles indicate three wells of the nanowell array that contained a single-cell and Panel B presents the corresponding position on the PVDF membrane. In panel C the position of the same-colored circles is highlighted in the scatterplot. As a result, the green circle indicates the high producer, the blue circle that of a medium producer, and the red circle that of an average producer. After the anti-Her2 IgG production is measured the nanowell plate is taken out of the incubator, placed on the Puncher and the selected cells of interest are isolated based on its Her-2 production according to the schematic of Fig. 1.

### Selection and expansion

After punching single CHO cells from the nanowell array into a well of a 96-well plate, the cell plus the remnants of the well bottom are present in the well of the 96-well plate. In approximately 27% the cells are still attached to the glass remnants of the well bottom after punching. The top and bottom row of supplementary Figure S1 shows brightfield images of a single CHO cell after 1, 3, 6, and 9 days of punching into a single well of a 96-well culture plate. In the top row, the cell moved away from the remnants of the nanowell bottom and in the bottom row, the cell remains attached to the remnants. The outgrowth of the cells did not seem to be affected by the cell being attached to the remnants of the bottom or not.

Optimization of the culture conditions is needed for single CHO cell expansion. Single-cell outgrowth efficiency after 5, 10, and 15 days of culture is illustrated in supplementary Figure S2 showing the significant improvement in cell outgrowth by adding InstiGRO (cell growth stimulation supplements) to the ProCHO-5 medium. After 15 days of culturing, 39% of the single cells grew to a large number of cells to create a clone (cell line) from the originally “punched” single CHO cell.

### Her-2 production CHO cells after single-cell isolation

**Figure 4:**
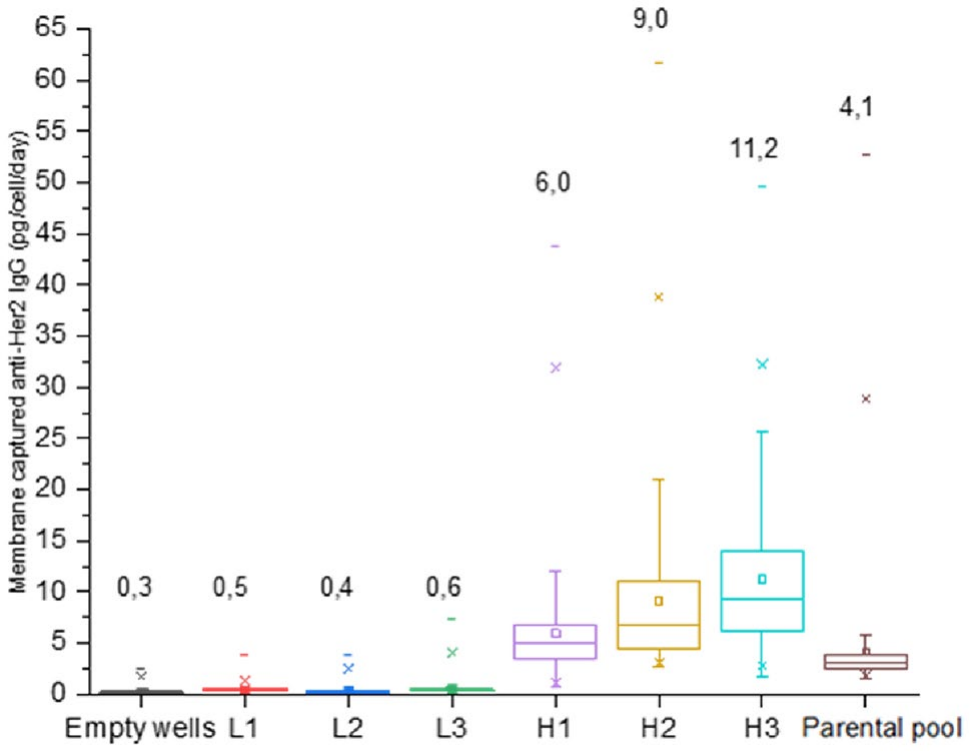
Her-2 production of of the individual cells of the different clones after printing the secreted Her2-IgG with the nanowell device. The cells were seeded in the microwells, and anti-Her2 IgG was detected on a PVDF membrane. The fluorescence signal intensity was used to quantify secreted antibody for low (L1-L3), high (H1–H3) and parental pool cells at the single-cell level. Empty wells represent the fluorescence background signal on the membrane for wells without a cell. The numbers above each clone show the average single cell antibody production in pg/(cell*day).

While we select high and low producer single cells, we expect to find higher but also lower levels of antibody production. To assess whether the selection of high Her-2-producing CHO cells could lead to subclones producing higher amounts of Her-2, low and high producers were identified, isolated, and expanded. From the isolated 54 high producers, 11 grew out to a stable cell line and from the isolated 16 low producers, 3 grew out to a stable cell line. To determine the average production of anti-Her2 IgG production at single-cell level 3 high producer and 3 low producer clones were sorted in the nanowell and the average anti-Her2 IgG production was measured on PVDF membranes. The Her-2 production from the parental pool and the production from the 3 low (L1, L2, L3) and 3 high (H1, H2, H3) producing CHO clones after 24 h of incubation is shown in Fig. 4. The average production of the parental pool was 4.1 pg/cell/day, and the averages of the single-cell antibody production of the 3 low producers of CHO cell clones were 0.45 (L1), 0.34 (L2) and 0.58 (L3) pg/cell/day and the averages of the 3 high producers CHO clones were 6.0 (H1), 9.0 (H2), 11.2 (H3) pg/cell/day indicating a ~ 2.3-fold increase in antibody production of the isolated high producing cells as compared to parental pool cells and a ~ 19-fold increase in antibody production as compared to the low producing CHO cells.

### Scale-up Her-2 production of selected CHO cell clones

**Figure 5:**
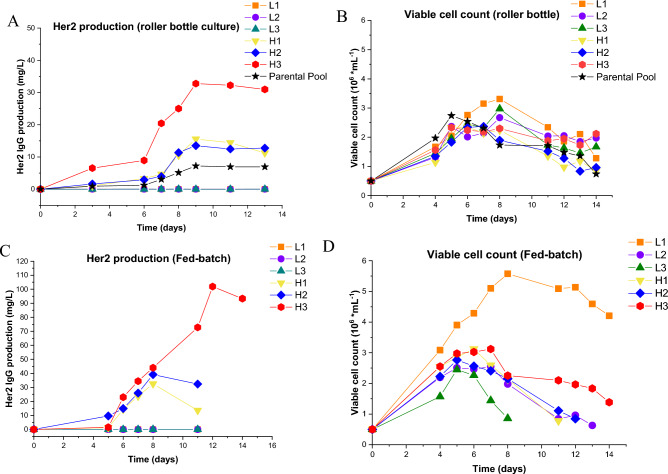
Anti-Her2 IgG concentration in the supernatant and the production in pg/cell/day of roller and FED batch cultures. (**A**) The anti-Her2 IgG concentration from roller bottles of the selected high-producer clones H1, H2, and H3, low producer clones L1, L2, and L3, and the parental pool from which these cells are derived. (**B**) The viable cell counts of the different clones at different time points in the roller bottle. (**C**) The concentration of anti-Her2 IgG in the supernatant of the Fed-batch culture for the high producers H1, H2, and H3 and low producers L1, L2, and L3. (**D**) The viable cell counts for the clones in the Fed batch at different time points.

To determine whether the increase in antibody production was sustained after expansion of the clones the clones were expanded in roller bottles and fed batch cultures. The amount of produced antibodies by individual cells from these cultures was determined by ELIspot. Figure 5A,C display the concentration of antibodies in the supernatant over a period of 1–14 days of the parental pool cells (cells which were not screened using the nanowell platform) and selected and expanded low and high-producing cells in the roller bottles and fed batch cultures. Figure 5B,D present the viable cell count for each clone during the assays for roller-bottles and fed-batch cultures respectively. In general, the number of viable cells is highest for the fed-batch cultures, especially clone L1. In general, it was expected that fast growing clones would produce low titers of Her2 IgG. Although this is true for clone L1 (fast growing and secreting low amounts of Her2 IgG) the data does not show a clear correlation between cell growth and antibody production. In the roller bottle culture clones showed a decrease in cell viability after 8 days in the roller bottle culture while in the fed-batch it was clone dependent. The results indicate that the higher antibody production of the high antibody-producing clones (H1, H2, and H3) was maintained in the bulk systems whereas the antibody production of the low antibody-producing clones (L1, L2, and L3) remained low. The supernatant of high-producing clone H3 in the fed batch culture (Fig. 5C) showed the highest titer 100 mg/L while the titers of clones H1 and H2 were 19 and 38 mg/L respectively. The antibody titers of the low producer clones L1, L2, and L3, were low or non-detectable indicating that our nanowell selection device could identify and separate the high IgG expressing cells from the low expressing cells at the single cell level.

## Discussion

Biopharmaceutical organizations are under increasing pressure to streamline their antibody discovery and cell line development processes, with unmet needs for increased throughput, shortened timelines, reduced costs, and more stringent requirements to prove monoclonality. Next-generation, single cell analysis platforms like our system, allow researchers to screen hundreds of thousands of individual cells, for the secreted target protein, to isolate high-value cells or pools of interest, and to dispense with high viability into 96-well plates.

A nanowell platform is introduced to rapidly screen, identify, and select high antibody producing single CHO cells to obtain clones through which antibody production is strongly improved. This nanowell screening platform accurately identifies and isolates individual cells using an efficient method of single cell seeding and isolating in combination with imaging and common ELISA methods. The nanowell platform is much more effective and efficient than traditional clone screening methods in three ways. First, this method assures monoclonality, as cells are sorted as individual cells in individual wells which is confirmed by imaging. Secondly, the nanowell device enables the selection of cells based on their IgG secretion at the single cell level without the need to culture, a feature that is not possible with limiting dilution approaches. Third, it uses common ELISA methodology to determine the production of antibodies and doesn’t require the development of additional reagents.

The nanowell device contains 6400 nanowell in an 8 × 8 mm^2^ in which typically 5000–6000 individual cells are present after cell seeding at a cell-loading concentration of 5000 cells/mL. Nanowell device also enables the use of lower concentrations of cells without affecting the number of cells seeded in the wells. Additionally, seeding the cells in the nanowell device is very efficient without the need for an expensive pipetting instrument and only requires approximately 20 min which is a large improvement compared to limiting dilution. A crucial factor behind this expedited process is the one-step dilution technique of the nanowell device, requiring only one serial dilution step to achieve a concentration of 5000 cells/mL. In contrast, the limiting dilution technique necessitates multiple serial dilutions ^15,16^. To be more precise, the exact concentration of cells is not critical but the total number in the sample volume needs to be approximately 5000 cells. Another large advantage of using the nanowell platform lies in the fact that it uses a limited amount of reagents and plastics. The small volume size of the wells, limits the use of reagents (1–2 ml per nanowell chip). Using limiting dilution requires a very diluted cell mixture. As a consequence, many wells on the 96 well plate end up empty, and only a few contain a single cell. This not only leads to a lot of wasted resources like reagents, media, and 96 well plates but also requires the processing of a large number of plates to get the desired number of single cell clones. Lastly, it requires at least two of limiting dilution and cloning to increase the chances of achieving monoclonality^25^. As a result, the whole process of single cell cloning and generating cell lines takes around 10–20 weeks for limiting dilution.

The nanowell platform presented here was able to establish anti-Her2 secreting clones from single cells that were isolated based on their antibody production. The increased production was maintained in roller bottles and Fed batch cultures confirming. This indicates that the platform has the potential to screen thousands of cells and establish mAb-secreting cells within 4–5 weeks without the need for multiple cloning steps.

The nanowell platform can identify and isolate IgG secreting cells at the single-cell level in 8–16 h, without the need to grow the cells for days. In addition, it offers the possibility to check the obtained microscopic images of each well to ensure monoclonality. Due to the short screening time, cells remain viable and can be retrieved from the well by punching into a collection plate. Once the antibody producing cells are identified (4 h), cells can be retrieved from the nanowell chip. Retrieval of 96 cells (single 96 wells plate) by punching the cell residing on the nanowell bottom will take 2–3 h (2 min/cell) which is the only challenge in the current protocol. This indicates that cells can die during the prolonged absence from the incubator.

We demonstrate for the first time a nanowell platform that can be used to 1. identify high producing antibody producing CHO cells; 2. Isolate these cells individually; 3. Create clonal CHO cells with high antibody production compared to their parental clone and 4. Maintain the high antibody production in FED batches. Compared to a traditional limiting-dilution workflow, the nanowell platform can efficiently shorten the screening and identification of production cell lines.

## Materials and methods

### Herceptin (Her-2) producing Chinese hamster ovary (CHO) cells

CHO cells producing Her2 were kindly provided by Polpharma (Polpharma Biologics B.V., Utrecht, The Netherlands). CHO cells were cultured in serum-free ProCHO medium (Lonza, Verviers, Belgium) supplemented with 2% pluronic F68 (Invitrogen, Carlsbad, USA) and 4mM L-glutamine. For passaging, suspension cultures were harvested by thorough pipetting, and cells were pelleted by centrifugation and resuspended in fresh complete medium. Subsequently, cell number and viability were determined by incubation of the cells with 4% Trypan blue (Invitrogen, Carlsbad, USA) and counted on a Luna-FL™ automated cell counter (Logos Biosystems**,** Westburg B.V., Leusden, the Netherlands)**.** Cells were cultured in T25 non-treated culture flasks (VWR international B.V, Amsterdam, the Netherlands) suitable for suspension cultures at a density of 2 × 10^5^ cells/mL in a humidified incubator at 37°C under 5% CO_2_.

### Antibodies

Anti-Her2 and anti-hIgG were purchased from Acrobiosystems (Newark, USA). Anti-human IgG-FITC (H + L) (Fab) was purchased from Sigma Aldrich (St Louis, USA).

### Preparation of the capture membranes

Low fluorescence Polyvinylidene membrane (PVDF) with a pore size of 0.45 was purchased from Bio-rad laboratories (California, USA). PVDF was cut to 1 × 1 cm dimensions and wetted in 100% methanol (Fisher Scientific, Leicestershire, UK) followed by 3 washes in sterile MilliQ. Next, PVDF membranes were coated with ligands, anti-hIgG (30µg/mL) or anti-Her2 (25ug/mL) overnight at 4 °C. Subsequently, membranes were washed in Phosphate buffered saline (PBS), (EMD Millipore, Milleroca, USA) and blocked in buffer consisting of 3% BSA (Sigma Aldrich, St Louis, USA) in PBS for 1h at RT. Finally, the PVDF membranes were washed once in PBS and incubated in cell culture medium before use.

### Preparation of nanowell arrays

Nanowell arrays and pump-holder (VYCAP B.V, Enschede, the Netherlands) were γ-sterilized (γ-irradiated). Nanowell were degassed by inserting in the pump-holder and adding sterile PBS on top of the nanowell chip and left for 30 min to allow PBS to enter the wells. Subsequently, before cells were added, fresh medium was filtered through by adding a small negative pressure of 5mBar to fill all wells with medium.

### Single-cell antibody printing

Cells were incubated with 1 mM of Calcein AM stain (Invitrogen, Carlsbad, USA) for 30 min or with CellTracker Orange (Invitrogen, Invitrogen, Carlsbad, USA) at a dilution of 1:5000 v/v for 60 min. Labeled cells were spun down and washed twice in PBS and once in complete culture medium to remove any secreted antibodies present in the supernatant. Next, a cell suspension containing around 6500 single cells suspended in complete medium was distributed into the nanowell array by applying a small negative pressure of 5–10 mbar. The nanowell arrays were imaged with an automated inverted epifluorescence microscope. The prepared PVDF capturing membrane was next sandwiched between the bottom of the nanowell arrays and a PDMS slab using a clamping device. Finally, the device was incubated at 37 °C and 5% CO_2_ for 24h.

### Detection of printed antibody arrays

For the detection of cell-secreted proteins, the PVDF membranes were washed once in PBS with 0.05% Tween 20 and PBS (5 min) to remove cell debris. Hereafter membranes were blocked in a blocking buffer for 1h at RT and incubated with PE-conjugated detection antibodies, anti-human IgG (1:1000 v/v) for 1h at RT. Finally, the membranes were briefly washed twice in PBS (5 min) and once in MilliQ and then dried. The membranes were imaged using an automated inverted epifluorescence microscope.

### Calibration curves

To convert the measured fluorescence intensity to concentrations of anti-Her2 drops of 1µl containing different concentrations of recombinant anti-Her2 (0–400µg/mL) were spotted on the PVDF membrane. The recombinant antibodies were reconstituted in complete culture medium (ProCHO-5 medium). The amount of antibody in each spot is known and dividing this by the area of the spot results in the antibody concentration in pg/µm^2^.

### Image analysis and quantification of fluorescent signal

Images of the nanowell array and fluorescently labeled PVDF membrane were acquired using the imaging function and software of the Puncher system (VYCAP). This system uses an LED excitation light source, a 20X, NA 0.45 objective, and a CMOS camera to acquire in total 10 × 10 images of the entire 8 × 8 mm surface of the nanowell chip and PVDF membrane. Images are stored in tiff format without any data compression or loss of signal. The fluorescent images were loaded into the software to quantify the total membrane-captured antibody spots. The location of the spots on the membrane identifies the nanowell number that contains the cell that secreted the Her2. An in-house software algorithm, using Labview’s IMAQ image analysis routines (National Instruments, Austin, Texas, USA), determined the location of the same nanowell on the different membrane measurements. The amount of captured Her2 was next determined by measuring intensity in each spot and comparing it with the generated calibration values.

### Single cell sorting and identification and isolation

After a cell suspension is placed onto a self-sorting nanowell device, the suspension passes through the pores at the bottom of the nanowells. Fluidic forces pull the cells towards the pore and as soon as it lands on the pore it stops the flow towards that particular well thereby avoiding that a second cell will enter the well. Next, the nanowell chip is positioned on an inverted fluorescence microscope to capture images of the bottoms of the nanowells, further verifying the presence of only one cell. The inverted microscope is equipped with two connected XY stages and a stage that holds a NiTi punch needle. After identification of the nanowells of interest, the NiTi needle is lowered into the nanowell and punches the well bottom containing the cell into a well of a 96-well plate. The end of the NiTi wire is shaped such that it only touches the bottom of the nanowell and not the captured cell. This Puncher system for cell imaging and cell retrieval (VYCAP, Enschede, the Netherlands) is described in detail elsewhere^22–24^.

#### Cell culture and FB procedure

Cell lines were cultured in a shake flask (SF, 125 mL format, Corning) on an orbital shaker (125 rpm, 2.5 cm pinch, Celltron, Infors) at 37 °C/5% CO2 humidified conditions. Cells were passed twice per week in ProCHO-5 medium (Lonza) supplemented with 4 mM L-glutamine (Gibco) and 0.1% Pluronic® F-68 (Gibco)at 0.5 × 10^6^ viable cells/mL. Starting from these cultures, the fed-batch (FB) evaluation test was initiated. The cells were seeded at 0.5 × 10^6^ viable cells/mL in fresh medium in a 125 mL SF format and incubated under the conditions described above. The FB cultures were monitored for 14 days on viable cell density, viability (LUNA™ cell counter, Logos Biosystems), glucose, and lactate concentration (Biosen S-Line, EKF Diagnostic). A flat feed procedure was followed from day 4 onwards. A daily volume of 2% (v/v) Cell Boost™ 7a and 0.2% (v/v) Cell Boost™ 7b (Cytiva) was added to the FB cultures. Additionally, a 4 g/L glucose concentration was maintained in the FB cultures by adding an additional glucose stock solution from day 4 onwards (Sigma). From day 5 onwards the recombinant protein expression levels were determined by Bio-Layer Interferometry (BLI) analysis (Octet RED96e, Sartorius) using protein A biosensors and an Ig standard as reference.

#### Cell culture and roller bottle procedure

For the roller bottle procedure, the cells were seeded at 0.5 × 10^6^ viable cells/mL in fresh medium ProCHO-5 medium (Lonza) supplemented with 4 mM L-glutamine (Gibco) and 0.1% Pluronic® F-68 (Gibco) in a 125 mL SF. The cultures were followed for 14 days on viable cell density (LUNA™ cell counter, Logos Biosystems). During the culture, 50 uL medium was removed from the cultures spun down and the supernatant was frozen at − 30 °C. For each time point the recombinant protein expression levels were determined by spotting 2 uL of medium (n = 4) harvested from the clones (high, and low selected with the nanowell platform, and the parental pool from which the cells were selected), and the spots visualized with Anti-human IgG-FITC and a Her2 IgG standard as reference was used to quantify amounts of cell secreted anti-Her2 IgG from the different clones.

### Supplementary Information

Supplementary Figures.

## Data Availability

The datasets used and/or analysed during the current study are available from the corresponding author upon reasonable request.
