# Supplementary material for: A nanowell platform to identify, sort and expand high antibody-producing cells
Source: Sci Rep. 2024 Apr 24;14:9457. doi: 10.1038/s41598-024-60054-1 (PMC11043069; doi:10.1038/s41598-024-60054-1)
Supplement: Supplementary file 1 — Supplementary Figures. [file 41598_2024_60054_MOESM1_ESM.docx]

## **Supplementary Figures**


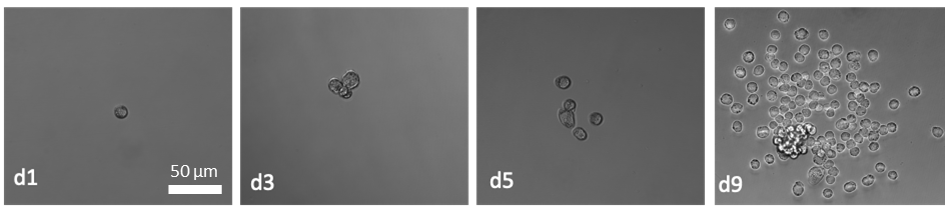


Figure S1: *Bright field images of a typical single CHO cell 1 day (d1), 3 days(d3), 6 days (d5), and 9 days (d9) after “punching” and culture.*

**
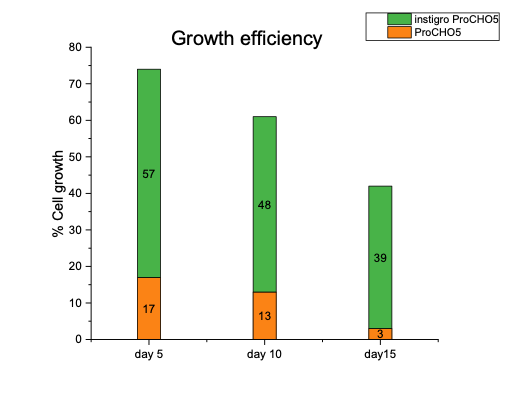
**

*Figure S2: Single cell CHO cell outgrowth after “punching” in ProCHO-5 medium and ProCHO-5 medium supplemented with InstiGRO. Cell growth of clones after 5, 10, and 15 days after punching. Single cell outgrowth in proCH0 5 medium supplemented with instiGRO resulted in 39% of the clones to grow into a cell line and in proCHO 5 medium 3 % of the clones proliferated into a cell line.*
